# Preoperative Differentiation of Non-Subungual Glomus Tumors from Other Superficial Soft Tissue Tumors Using a Clinical and Ultrasound-Based Model

**DOI:** 10.3390/biomedicines13122883

**Published:** 2025-11-26

**Authors:** Hongjin Xiang, Qing Dan, Yue Zhai, Anran Guo, Yuzhou Shen, Run Wang, Desheng Sun, Xiangmei Chen

**Affiliations:** Department of Ultrasound, Peking University Shenzhen Hospital, Shenzhen Peking University-The Hong Kong University of Science and Technology Medical Center, Shenzhen 518036, China; hilay.shanon@foxmail.com (H.X.); qingdan@pkuszh.com (Q.D.); zzzzhaiyue@163.com (Y.Z.); guoanran1230@163.com (A.G.); cindysyz@163.com (Y.S.); wr9430@163.com (R.W.)

**Keywords:** non-subungual glomus tumors, soft tissues, ultrasonography, vascular stalk sign, logistic regression

## Abstract

**Objectives:** Non-subungual glomus tumors (NSGTs) are rare neoplasms arising outside the nail bed, typically presenting with disproportionate pain. Surgical excision usually achieves complete relief. Delayed or incorrect diagnosis of glomus tumors may also result in incomplete excision, which is the major cause of recurrence. Ultrasound is a well-recognized tool for preoperative evaluation, but the rarity of NSGTs complicates differentiation from angioleiomyomas (ALMs) and hemangiomas, which have alternative managements. This study aimed to establish an ultrasound diagnostic model and scoring system for NSGTs. **Methods:** A total of 58 NSGTs, 68 ALMs, and 67 hemangiomas confirmed pathologically between January 2014 and March 2025 were analyzed. Clinical and ultrasonographic features were evaluated, and significant variables were incorporated into a scoring time using the binary logistic regression. A total of 11 NSGTs, 10 ALMs, and 10 hemangiomas (April–August 2025) were used to validate the diagnostic performance of the scoring system. **Results:** Four independent predictors were identified: pain, upper extremity location, diameter < 10 mm, and vascular stalk sign (*p* < 0.05). The model differentiated NSGTs from ALMs and hemangiomas, yielding an AUC of 0.91, with sensitivity of 75.86% and specificity of 90.37%. The overall accuracy was 86.01%. **Conclusions:** Ultrasound proves valuable in diagnosing NSGTs. A logistic regression model based on pain, location, size, and vascular stalk sign shows high diagnostic performance and clinical utility in distinguishing NSGTs from other superficial soft tissue tumors.

## 1. Introduction

Glomus tumors (GTs) are rare neoplasms that arise from the glomus body, typically composed of glomus cells, smooth muscle cells, and vasculature, which account for less than 2% of all soft tissue tumors [1]. GTs can be classified into subungual and non-subungual categories based on their anatomical location. Subungual GTs specifically occur beneath the nail, while non-subungual GTs (NSGTs) occur in various other soft tissues, including the head and neck [2], trunk [3], or even deeply (bone [4], trachea [5], breast [6], lung [7], stomach [8], kidney [9], prostate [10], etc.). The prevalence of NSGTs are poorly defined, representing about 30% of all GTs [11].

In typical cases of subungual GTs, clinical presentations with a triad of pain, tenderness, and cold hypersensitivity make them a common consideration for differential diagnoses in patients with neoplasms [12]. However, many NSGTs remain misdiagnosed or delay-diagnosed for months or years, particularly in cases at diverse locations, or if they have vague symptoms or similar presentation to other superficial soft tissue lesions, such as angioleiomyomas (ALMs) and hemangioma. Less than 20% of NSGTs are correctly diagnosed preoperatively [11], making prompt and complete excision both diagnostic and therapeutic [13,14,15]. Delayed or incorrect diagnosis of glomus tumors may also result in incomplete excision [16,17]. Incomplete excision is the major cause of recurrence, with historical recurrence rates as high as 20–33% [16]. Conversely, misdiagnosis as ALM may lead to excessive tissue removal, given that ALM has a low but real malignant potential [18]. Therefore, early identification of NSGTs allows precise surgical planning, complete excision, prevention of recurrence, and avoidance of unnecessary diagnostic interventions (e.g., MRI, nerve conduction studies, and exploratory surgery) [16,19].

Ultrasonography is being used extensively in the assessment of soft tissue masses [20], usually presenting hypoechoic on gray-scale ultrasound. Despite advances in imaging modalities and increased awareness among healthcare providers, several unresolved problems persist regarding NSGTs, such as the variability in clinical presentation that complicates early detection, and the lack of consensus on the most effective imaging features for diagnosis.

Given that previous publications [11,16,19,21,22,23,24,25] offered only limited descriptions of the clinical and ultrasonographic features of NSGTs, this study retrospectively analyzed a large cohort of patients over a 10-year period to comprehensively demonstrate these clinical and ultrasonographic findings, differentiating from ALMs, and hemangiomas—conditions that are often misdiagnosed in clinical practice. Our study intends to establish an ultrasound diagnostic model by logistic regression analysis, improving the management of NSGTs.

## 2. Materials and Methods

### 2.1. Inclusion and Exclusion Criteria

This study included patients in our hospital who were histopathologically diagnosed with NSGTs, ALMs, and hemangiomas from January 2014 to August 2025. Data from January 2014 to March 2025 were used for logistic regression model development and data from April 2025 to August 2025 were used for model performance validation. Exclusion criteria were: (1) patients with sublingual GT; (2) patients underwent treatments for NSGTs, ALMs, and hemangiomas; and (3) patients complicated with other vascular anomalies; (4) cases with poor ultrasound image quality (including low resolution, unproper gain settings, incorrect focal zone or position, and presence of artifacts). Due to the rarity of NSGTs and ALMs, all patients met the inclusion criteria were included in this study, while hemangioma were consecutively included in the same period. This study was approved by the Ethics Review Committee of our institute, and the informed consent was waived because of the retrospective nature of the study (No. 2024178).

### 2.2. Data Collection

The following demographic and clinical data were gathered: age, sex, symptoms, mass site, and mass size. Telephone follow-up was performed for all patients with NSGTs.

### 2.3. Ultrasonography Examinations

All images were retrospectively retrieved from a database through a consensus of two highly trained musculoskeletal sonographers, with 12 and 10 years of experience, respectively. All lesions were examined using high-frequency transducers with a frequency of 13 MHz or higher. Gray-scale images were obtained in both transverse and longitudinal orientations. Color Doppler settings were optimized to maximize sensitivity to low-velocity flow, including a velocity scale of 1.8–7 cm/s, low wall filter, and optimized Doppler gain adjusted just below the noise threshold.

### 2.4. Images Analysis

In the gray-scale sonography data, several key characteristics were documented, including mass size, shape, orientation, margins, echogenicity, echo texture, vascularity grading, and presence of vascular stalk sign. Size was measured as the maximum diameter, encompassing the lesion’s length and thickness. Shape was classified into three categories: oval, round, or irregular. Orientation was noted as either distinctly vertical or horizontal, reflecting the relationship between the skin surface and the lesion’s major diameter. Margins were defined as well-circumscribed when they were clear all around the lesion and ill-circumscribed when there was any degree of blurring at any segment of the lesion’s border. Echogenicity was categorized as hypoechoic, isoechoic, or hyperechoic in comparison to the surrounding perilesional tissue. The echotexture was classified into homogeneous or heterogeneous, based on the variation in echo reflection. Regarding color Doppler imaging, the vascularity was defined as: grade 0, no flow detected; grade 1, 1–4 dot-like color signals; grade 2, 1 or 2 linear color signals or ≥5 color dots; or grade 3, ≥3 linear color signals. In addition, the presence of vascular stalk sign was documented, which was characterized by a hypoechoic area connecting the lesion to the adjacent soft tissue, showing intense color Doppler signal and reflecting the vascular origin [22].

### 2.5. Statistical Analysis

Statistical analyses were conducted using SPSS 28.0 (IBM^®^ SPSS Statistics, Armonk, NY, USA) and PRIMS GraphPad version 10.0 software (GraphPad Software, Boston, MA, USA). Continuous variables (e.g., age, lesion length, thickness, and length/thickness ratio) were expressed as mean ± standard deviation or median (interquartile range) as appropriate, and were compared between NSGTs and ALMs, and between NSGTs and hemangiomas using the Mann–Whitney U test. Categorical variables, including sex, symptoms, mass location, shape, margin, echogenicity, echotexture, vascularity grade, and the presence of a vascular stalk sign, were compared using Chi-square tests or Fisher’s exact tests when the expected cell count was <5. The significantly different variables were selected, and the backward stepwise selection method was used to carry out multivariate binary logistic regression analysis. The dichotomous forms of maximum lesion diameter (<10 mm vs. ≥10 mm) and mass length/thick ratio (<2 vs. ≥2) were used in the logistic regression model. Receiver operating characteristic (ROC) analysis was used to evaluate the diagnostic performance of the logistic regression model by calculating the area under curve (AUC). The Youden index was applied to identify the optimal cut-off, sensitivity and specificity for the logistic regression model. The overall accuracy was derived from the confusion matrix at this cut-off. A *p*-value of <0.05 was considered statistically significant. 11 NSGTs, 10 ALMs, and 10 hemangiomas from April 2025 to August 2025 were used to validate the model performance.

## 3. Results

### 3.1. Patient Characteristics

This study included 58, 68, and 67 patients who were pathologically confirmed with NSGTs, ALMs, and hemangiomas, respectively, between January 2014 and March 2025. A total of 193 solitary lesions were identified. As summarized in Table 1, age and sex were not statistically significant between NSGTs and other superficial soft tissue lesions (ALMs and hemangiomas). Unlike the typical symptoms of subungual glomus tumors, the most common presentation of NSGTs were pain (53.45%) and tenderness (41.38%), with none of the patients reporting cold hypersensitivity. Less pain (19.12%) and tenderness (30.88%) were reported in ALM patients, and there was a significant difference between NSGTs and ALMs (*p* < 0.001). Also, hemangioma patients often presented significantly slight (*p* < 0.001) symptoms, with 4.48% pain and 19.40% tenderness. No recurrence was detected among the patients with NSGTs who were successfully followed (46/58). The follow-up duration was 48 (10,110) months.

### 3.2. Mass Site

As shown in Table 1 and Table A1, NSGTs were found at in various sites, including the extremities and the body trunk. Interestingly, it predominantly occurred in upper extremities (82.76%), with most (67.24%) in the fingers (*p* < 0.001). In contrast, 80.88% of ALMs developed at lower extremities, including the foot, calf, knee, ankle, thigh, and toe. Unlike NSGTs, hemangiomas could occur more frequently in the head, neck, and body trunk (*p* < 0.001).

### 3.3. Mass Size

The median diameter of an NSGT was 5 (3, 8.75) mm. In contrast, both ALMs and hemangiomas were greater in median diameter than NSGTs, which were measured as 10 (7.10, 17) mm and 15 (10, 31) mm, respectively. There is a significant difference in length among NSGTs, ALMs, and hemangiomas (*p* < 0.001) (Table 1). The median length/thickness ratio for NSGT patients was 1.78 (1.33, 2). ALM and hemangioma are greater than 2 and 2.5 (*p* < 0.001).

### 3.4. Sonographic Findings

#### 3.4.1. Shape and Orientation

**Figure 1:**
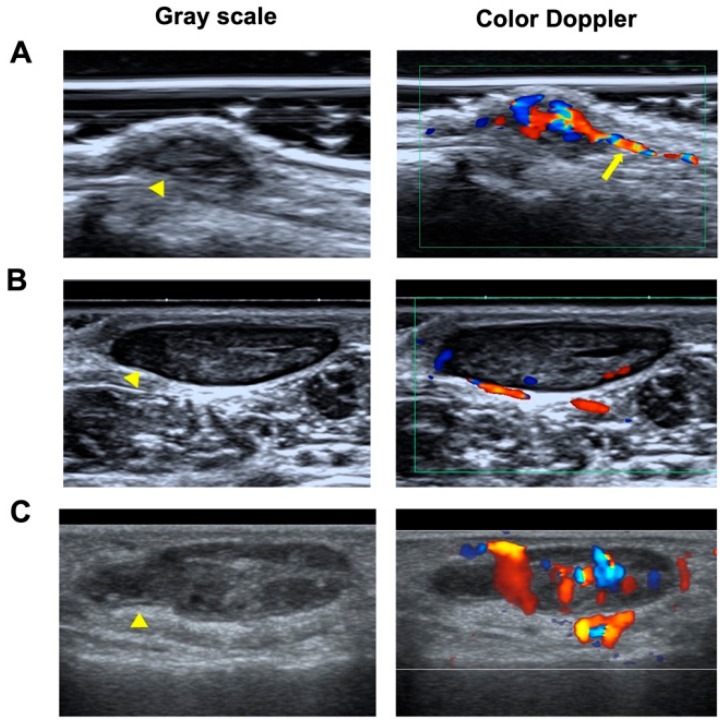
Representative gray scale and color Doppler ultrasonograms of NSGT, ALM, and hemangioma. (**A**) Gray-scale ultrasound images obtained using high-frequency ultrasound show a hypoechoic mass measuring 7 × 3 mm in the subcutaneous tissue in the right arm of a 56-year-old man (yellow arrowhead). Color Doppler ultrasound reveals a hypervascular lesion (grade 3) with a vascular stalk sign (yellow arrow). (**B**) A hypoechoic mass measuring 15 × 6 mm in the left foot of a 31-year-old man (yellow arrowhead), with vascularity of grade 1. (**C**) A hypoechoic mass measuring 19 × 6 mm on the right finger of a 36-year-old woman (yellow arrowhead), with vascularity of grade 3.

The sonographic features of NSGTs, ALMs, and hemangiomas were summarized in Table 2 and representative sonograms of the three types of diseased were shown in Figure 1. Most of NSGTs (75.86%) and ALMs (94.12%) presented an oval shape. Comparably, 49.25% of hemangiomas presented as irregular, significantly higher than their NSGT and ALM counterparts (*p* < 0.001). Nearly all the three types of tumors were horizontally orientated, with only one NSGT and one ALM in vertical orientation.

#### 3.4.2. Margin

NSGTs predominantly showed clear margins. Fifty-two (89.66%) well-circumscribed tumors and six (10.34%) ill-circumscribed tumors were observed in NSGT patients. Similarly, 64 (94.12%) ALM tumors were well-circumscribed and four (5.88%) ALM tumors were ill-circumscribed. In contrast, ill-circumscribed tumors accounted for a significantly higher proportion (50.75%) in hemangiomas (*p* < 0.001).

#### 3.4.3. Echogenicity and Echotexture

All NSGTs presented as hypoechoic. Similarly, 67 (98.53%) ALMs appeared as hypoechoic, with only one case in isoechogenicity. Most hemangiomas (92.53%) were hypoechoic, with three (4.48%) in isoechogenicity and two (2.99%) in hyperechogenicity. The internal echo of NSGTs was homogeneous in 51 (87.93%) patients and heterogeneous in seven (12.07%) patients. ALM appeared slightly higher in the portion (22.06%) of heterogeneous tumors, while it was not significantly different from NSGTs (*p* = 0.14). Hemangiomas were more likely to present as heterogeneous (76.12%) (*p* < 0.001).

#### 3.4.4. Vascularity

The blood vessel patterns observed by color Doppler ultrasound of these three types of superficial soft tumors could present as no, minimal, moderate, and marked blood-flow signals, with no significant differences (*p* > 0.05). Interestingly, as reported by Hee-Jin Park et al. [22], vascular stalk sign, reflecting the vascular origin of the tumor (Figure 1A), was observed in 38 NSGT patients (65.52%), significantly higher than that in ALMs (27.94%, *p* < 0.001) and hemangiomas (16.42%, *p* < 0.001).

**Figure A1:**
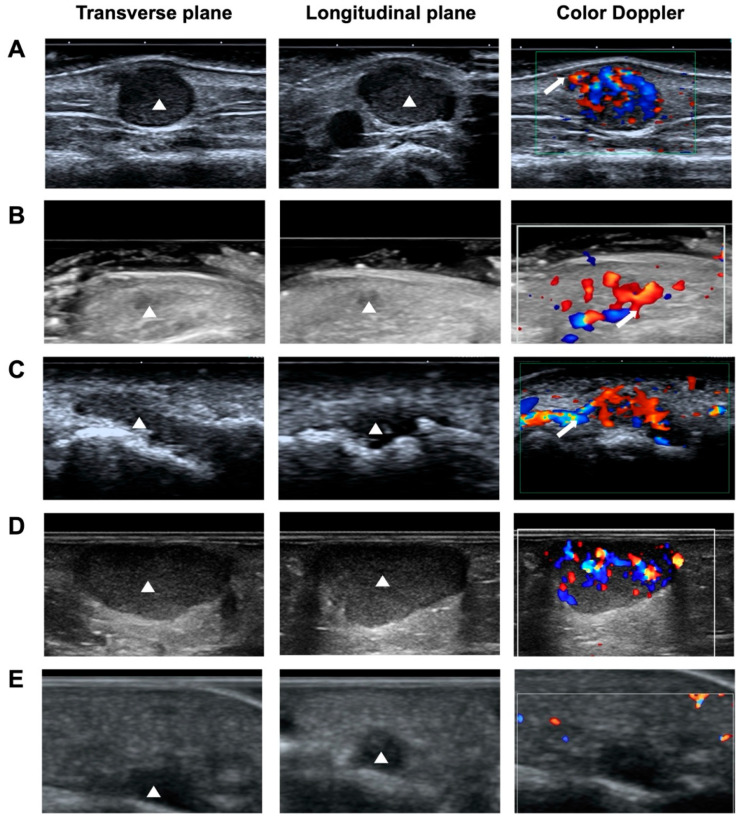
Ultrasound features of NSGTs. (**A**) NSGT appears as oval-shape, hypoechogenic, well-circumscribed, and hypervascular (with vascular stalk sign). (**B**) NSGT appears as irregular, hypoechogenic, ill-circumscribed, hypervascular (with vascular stalk sign). (**C**) NSGT appears as irregular, hypoechogenic, well-circumscribed, hypervascular (with vascular stalk sign). (**D**) NSGT appearss as oval-shape, hypoechogenic, well-circumscribed, hypervascular (without vascular stalk sign). (**E**) NSGT appears as irregular, hypoechogenic, ill-circumscribed, avascular (without vascular stalk sign).

In summary, a typical NSGT can present as pain and tenderness. It usually appears as a small, hypoechogenic, oval-shape, well-circumscribed lesion with vascular stalk sign on gray-scale ultrasound and color Doppler ultrasound. Other ultrasonographic characteristics can be seen in Figure A1. However, there are many common features between NSGTs and ALMs and hemangiomas. A logistic regression study was further conducted to identify the significant clinical and ultrasonographic features to improve the diagnostic performance of NSGTs when differentiating from other hypoechoic lesions on ultrasound scanning.

#### 3.4.5. Logistic Regression Study

**Figure 2:**
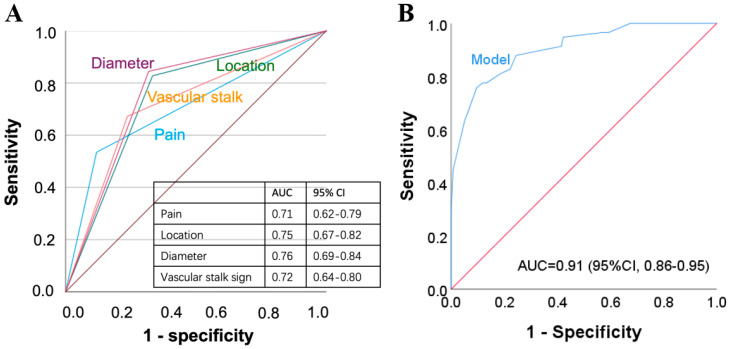
AUCs of four independent risk factors (**A**) and the predictive model that combined the four independent risk factors (**B**).

Univariate analysis showed that there were statistically significant differences in pain, tenderness, mass location, mass maximal diameter, mass length/thickness ratio, shape, margin, and vascular stalk sign (Table 1). Four criteria (pain, mass location, mass diameter, and vascular stalk sign) were found to be independent risk factors of NSGTs (*p* < 0.05) (Table A2). ROC curves and AUCs of pain, mass location, mass diameter, and vascular stalk sign were shown in Figure 2A, respectively. The AUC with 95% CI for each variate was presented. Since no single individual variate was found to be satisfactory for predicting NSGT in terms of the AUC, we further established a model that used the results obtained for each variate.

The fitted model was established: Logit(*p*) = 0.03 + 5.14 × X_1_ + 10.20 × X_2_ + 7.13 × X_3_ + 4.38 × X_4_. Where X_1_ indicates the pain (presence of pain = 1, absence of pain = 0); X_2_ indicates the mass location (upper extremities = 1, other locations = 0); X_3_ indicates the mass (<10 mm = 1, ≥10 mm = 0); and X_4_ indicates the vascular stalk sign (presence of vascular stalk sign = 1, absence of vascular stalk sign = 0). The AUC for using this formula to diagnose NSGTs was 0.91 (Figure 2B). The cut-off, sensitivity, and specificity were 17.02%, 75.86%, and 90.37%, respectively. The overall accuracy was 86.01%. In the validation cohort, the diagnostic scoring system has a validated accuracy of 83.87% (26/31).

## 4. Discussion

NSGTs present significant diagnostic challenges due to their rarity, atypical locations, and uncommon clinical manifestations, often leading to prolonged diagnostic delays or misdiagnoses of these lesions. Even if the clinical diagnosis of a NSGT is correct, conducting a surgical operation without exact preoperative imaging evaluation of the site and size of the tumor may result in incomplete excision, thereby leading to recurrence [19]. High-frequency ultrasound has been well-recognized as the first tool of diagnosis and evaluation in GTs. It allows for accurate planning of the excision by precisely establishing the size, location, and vascularization of the tumor, especially for non-palpable lesions. However, we found that only a minority of the prior publications involved sonographic appearances of NSGTs from small study cohort, collecting four [26], six [21], nine [22], 10 [25], 18 [11], and 19 [19] cases of NSGTs, respectively. To the best of our current knowledge, our study, which encompasses the largest cohort of 58 NSGT patients, represents the first logistic regression model aimed at improving the diagnostic efficacy and efficiency for this condition.

In this study, we used 11 clinical and ultrasonographic characteristics to select the significant features and develop the model to diagnose NSGTs. Age, sex, and sonographic findings including shape, orientation, echogenicity, echotexture, and vascularity did not show significant differences (*p* > 0.05). We found that the presence of pain, location at upper extremity, diameter < 10 mm, and presence of vascular stalk sign on color Doppler were independent risk factors in the differentiation of NSGTs, ALMs and hemangiomas.

The AUCs of these four factors were 0.71 (pain), 0.75 (location in upper extremity), 0.76 (diameter < 10 mm), and 0.72 (presence of vascular stalk sign). Since in clinical practice, no diagnosis can be made through single feature, a combination of useful clinical and ultrasonographic features via a binary logistic model provided better diagnostic accuracy, with an AUC reaching 0.91. The sensitivity, specificity, and accuracy were 75.86%, 90.37%, and 86.01%, respectively.

This study found that the most common clinical presentation of NSGTs was pain, which was reported in 53.45% of our cases. However, ALMs and hemangiomas reported significantly less pain. This indicates that NSGTs pose a negative impact on patients’ lives despite being a benign tumor.

Location is a valuable discriminator for identifying NSGTs. Glomus bodies are predominantly located in the dermis, with high concentrations in the fingertips, nail beds, palms, and soles [27]. In our cohort, NSGTs overwhelmingly arise in the upper extremities (82.76%), with a particularly high prevalence in the fingers (67.24%). A clinical review of 42 extradigital GTs reported that 59.52% occurred in the upper extremities [23]. By contrast, Orlando et al. documented a site predominance in long segments of the extremities (thigh, leg, forearm) in a cohort of 18 patients [11]. These discrepancies likely reflect small sample size bias in earlier studies. Our study, the largest NSGT sample to date, demonstrates that a mass located in the upper extremity confers a high probability of NSGT, especially when accompanied by other features such as pain, small size, and the vascular stalk sign. Logistic regression confirms this location-based indicator, underscoring its potential clinical utility.

The size of the NSGTs (<10 mm) is an indicator helping differentiate between NSGTs, ALMs, and hemangiomas. Atypically, we also documented a large lesion on the knee with a size of 24 mm. Contrarily, ALMs and hemangiomas have larger median sizes in length of 10 mm and 15 mm, respectively.

On gray-scale ultrasound, an NSGT lesion typically appears as an oval-shape, horizontally oriented, well-circumscribed, and usually homogeneously hypoechoic nodule. On color Doppler ultrasound, although they can be avascular (18.97%), NSGTs were usually found to be vascularized to a variable degree (minimal, mild, marked). However, these findings were not significantly different from other soft tissue tumors.

Unlike hemangiomas, whose vascularity often presents as vascular lakes [22], NSGTs exhibit a distinct vascular pattern driven by their unique microvasculature. Histopathologically, glomus tumors are composed of nests of glomus cells surrounding irregular arteriovenous shunts. These arteriovenous anastomoses typically form between a preterminal arteriole and an efferent vein, often organized around a central feeding artery that courses from the periphery toward the tumor. This architectural arrangement creates a direct arterial input into the lesion with limited intervening capillary beds, producing a high-flow inflow that is detectable on color Doppler—the so-called vascular stalk sign. Our observational data show that the vascular stalk sign is present in 65.52% of NSGT cases, a finding that aligns with several prior investigations [11,21,22,25,26]. Importantly, the presence of this sign supports the diagnostic impression of NSGT when correlated with clinical features (presence of pain) and other imaging characteristics (e.g., small size, hypoechoic, well-defined, and hypervascular). Conversely, its absence does not exclude NSGTs, given the known variability in vascular architecture across lesions. Taken together, the vascular stalk sign serves as a highly specific sonographic indicator when observed, complementing a constellation of sonographic features and reinforcing the vascular pathology underlying NSGTs. This sign was validated by our diagnostic model, underscoring its potential utility in routine ultrasound assessment of suspected NSGTs.

Based on the satisfactory model performance, a clinical decision process is established to optimize the diagnosis of NSGT when ultrasound reveals a hypoechoic superficial soft-tissue mass. The approach combines clinical presentation and sonographic features to classify NSGT. Since, in the fitted model formula, mass location has a higher coefficient than pain, diameter < 10 mm, and vascular stalk sign, the location in an upper extremity has higher scoring points in the diagnostic scoring system. In the validation cohort, the diagnostic scoring system has a validated accuracy of 83.87%, which is superior to the accuracy in clinical practice, where no consensus was reached on NSGTs.

However, this study was subject to several limitations, primarily stemming from the inherent challenges associated with the rarity of the NSGT, which lead to a relatively small sample size. In this setting, a single-center retrospective study was conducted instead. Therefore, the follow-up duration had a wide range. Another limitation is the diagnostic scoring system, derived from a specific study population in a single center; external validation from multiple centers is essential. Diameter threshold may not capture atypical presentations (larger NSGTs) and could yield false negatives. The vascular stalk sign on color Doppler is a key component, but its detection is highly dependent on the ultrasound operator’s experience and the machine’s settings. Additionally, the scoring system may not account for comorbid conditions (e.g., vascular anomalies) that can confound ultrasound findings. Future work will include a prospective cohort from multiple centers, color Doppler ultrasonography with a standard setting, and systematically scheduled follow-up.

## 5. Conclusions

This study, with the largest NSGT samples to date, reveals that ultrasound proves to be valuable in diagnosing NSGTs. A typical NSGT can present as a painful, small, hypoechogenic lesion with vascular stalk sign in an upper extremity. The logistic regression model based on the presence of pain, mass location, mass diameter, and presence of vascular stalk sign has potential clinical value in the diagnosis of NSGTs.

## Figures and Tables

**Table 1:** Clinical Characteristics of Patients with NSGTs, ALMs, and Hemangiomas.

	NSGT (*n* = 58)	ALM (*n* = 68)	^a^ *p*	Hemangioma (*n* = 67)	^b^ *p*
Age (year)	40.98 ± 13.48	45.13 ± 13.23	0.11	38.99 ± 14.47	0.42
Sex (*n*, %)			0.66		0.98
Female	33 (56.90%)	36 (52.94%)		38 (56.72%)	
Male	25 (43.1%)	32 (47.06%)		29 (43.28%)	
Symptom (*n*, %)			<0.001		<0.001
Pain	31 (53.45%)	13 (19.12%)		3 (4.48%)	
Tenderness	24 (41.38%)	21 (30.88%)		11 (39.40%)	
Mass site			<0.001		<0.001
Head and neck	0 (0%)	1 (1.47%)		15 (22.39%)	
Upper extremity (*n*, %)	48 (82.76%)	10 (14.71%)		34 (50.75%)	
Lower extremity (*n*, %)	8 (13.79%)	55 (80.88%)		10 (14.92%)	
Trunk (*n*, %)	2 (3.45%)	2 (2.94%)		8 (11.94%)	
Mass size (median, IQR)					
Length (mm)	5 (3, 8.75)	10 (7.10, 17)	<0.001	15 (10, 31)	<0.001
Thickness (mm)	3 (2, 4)	5.65 (4, 7)	<0.001	6 (3, 10)	<0.001
Length/Thickness ratio	1.78 (1.33, 2)	2.23 (1.67, 3.29)	<0.001	2.75 (2, 3.71)	<0.001

^a^ *p*: NSGT vs. ALM; ^b^ *p*: NSGT vs. Hemangioma.

**Table 2:** Summary of Sonographic Findings.

Sonographic Appearance	NSGT	ALM	^a^ *p*	Hemangioma	^b^ *p*
Shape (*n*, %)			<0.001		<0.001
Round	6 (10.34%)	1 (1.47%)		0	
Oval	44 (75.86%)	64 (94.12%)		34 (50.75%)	
Irregular	8 (13.80%)	3 (4.41%)		33 (49.25%)	
Orientation (*n*, %)			>0.99		0.46
Horizontal	57 (98.28%)	67 (98.53%)		67 (100%)	
Vertical	1 (1.72%)	1 (1.47%)		0 (0%)	
Margin (*n*, %)			0.51		<0.001
Well-circumscribed	52 (89.66%)	64 (94.12%)		33 (49.25%)	
Ill-circumscribed	6 (10.34%)	4 (5.88%)		34 (50.75%)	
Echogenicity (*n*, %)			>0.99		0.25
Hypoechoic	58 (100%)	67 (98.53%)		62 (92.53%)	
Isoechoic	0 (0%)	1 (1.47%)		3 (4.48%)	
Hyperechoic	0 (0%)	0 (0%)		2 (2.99%)	
Echotexture (*n*, %)			0.14		<0.001
Homogeneous	51 (87.93%)	53 (77.94%)		16 (23.88%)	
Heterogeneous	7 (12.07%)	15 (22.06%)		51 (76.12%)	
Vascularity (*n*, %)			0.80		0.26
Absent	11 (18.97%)	18 (26.47%)		7 (10.45%)	
Minimal	11 (18.97%)	12 (17.65%)		18 (26.87%)	
Moderate	18 (31.03%)	19 (27.94%)		27 (40.30%)	
Marked	18 (31.03%)	19 (27.94%)		15 (22.38%)	
Vascular stalk (*n*, %)			<0.001		<0.001
Absent	20 (34.48%)	49 (72.06%)		56 (83.58%)	
Present	38 (65.52%)	19 (27.94%)		11 (16.42%)	

^a^ *p*: NSGT vs. ALM; ^b^ *p*: NSGT vs. Hemangioma.

## Data Availability

The data presented in this study are available on request from the corresponding author due to patient privacy and ethical restrictions.

## Abbreviations

The following abbreviations are used in this manuscript:

GTs	Glomus tumors
NSGTs	Non-subungual glomus tumors
ALMs	Angioleiomyomas
ROC	Receiver operating characteristic
AUC	Area under curve

## Appendix A

**Table A1:** Mass location of NSGT, ALM, and hemangioma.

	NSGT (*n* = 58)	ALM (*n* = 68)	Hemangioma (*n* = 67)
Mass location	Finger (39)	Ear (1)	Neck (5)
	Hand (5)	Finger (5)	Forehead (3)
	Arm (3)	Hand (2)	Face (3)
	Shoulder (1)	Wrist (2)	Eyelid (1)
	Teo (3)	Elbow (1)	Nose (1)
	Thigh (2)	Foot (21)	Lip (1)
	Knee (2)	Calf (11)	Head (1)
	Foot (1)	Knee (11)	Finger (12)
	Buttock (1)	Thigh (3)	Arm (11)
	Chest wall (1)	Ankle (6)	Hand (5)
		Teo (3)	Wrist (3)
		Epididymis (1)	Elbow (1)
		Buttock (1)	Shoulder (1)
			Axilla (1)
			Thigh (2)
			Knee (2)
			Popliteal fossa (1)
			Calf (1)
			Foot (3)
			Teo (1)
			Chest wall (1)
			Abdominal wall (1)
			Back (1)
			Waist (1)
			Buttock (1)
			Vulvae (3)
			Ectopectoralis (1)

**Table A2:** Backward stepwise logistic regression analysis. Pain, mass location, mass diameter, and vascular stalk sign were found to be independent risk factors of NSGTs.

		*p*	Exp(*B*)	EXP(*B*) 95% IC	
				Upper Bound	Lower Bound
Step 1	Pain (1)	0.003	4.923	1.694	14.305
	Tenderness (1)	0.082	2.534	0.887	7.239
	Location (1)	<0.001	10.415	3.672	29.547
	Diameter (1)	<0.001	6.534	2.296	18.590
	Length/Thickness ratio (1)	0.443	1.482	0.542	4.055
	Shape (1)	0.567	0.679	0.181	2.551
	Margin (1)	0.053	5.124	0.977	26.878
	Vascular stalk sign (1)	0.003	4.098	1.603	10.477
	Constant	<0.001	0.023		
Step 2	Pain (1)	0.003	4.875	1.686	14.096
	Tenderness (1)	0.084	2.514	0.885	7.139
	Location (1)	<0.001	10.733	3.805	30.271
	Diameter (1)	<0.001	6.267	2.230	17.611
	Length/Thickness (1)	0.408	1.525	0.561	4.144
	Margin (1)	0.057	4.128	0.961	17.721
	Vascular stalk sign (1)	0.004	4.004	1.576	10.172
	Constant	<0.001	0.023		
Step 3	Pain (1)	0.002	5.135	1.804	14.618
	Tenderness (1)	0.091	2.452	0.867	6.934
	Location (1)	<0.001	10.195	3.653	28.448
	Diameter (1)	<0.001	7.133	2.658	19.146
	Margin (1)	0.072	3.711	0.888	15.505
	Vascular stalk sign (1)	0.001	4.381	1.765	10.878
	Constant	<0.001	0.026		
