# Correction: Xiang et al. Preoperative Differentiation of Non-Subungual Glomus Tumors from Other Superficial Soft Tissue Tumors Using a Clinical and Ultrasound-Based Model. *Biomedicines* 2025, *13*, 2883

**DOI:** 10.3390/biomedicines14030708

**Published:** 2026-03-19

**Authors:** Hongjin Xiang, Qing Dan, Yue Zhai, Anran Guo, Yuzhou Shen, Run Wang, Desheng Sun, Xiangmei Chen

**Affiliations:** Department of Ultrasound, Peking University Shenzhen Hospital, Shenzhen Peking University-The Hong Kong University of Science and Technology Medical Center, Shenzhen 518036, China

**Error in Affiliation(s)**

In the published article [1], there was an error regarding the affiliations for all authors. The original affiliations 1 (Department of Ultrasound, Peking University Shenzhen Hospital, Shenzhen 518036, China) and 2 (Shenzhen Key Laboratory for Drug Addiction and Medication Safety, Shenzhen Peking University-The Hong Kong University of Science and Technology Medical Center, Shenzhen 518036, China) have been merged into one unified address. The correct affiliation is: Department of Ultrasound, Peking University Shenzhen Hospital, Shenzhen Peking University-The Hong Kong University of Science and Technology Medical Center, Shenzhen 518036, China. The authors state that the scientific conclusions are unaffected. This correction was approved by the Academic Editor. The original publication has also been updated.
